# Advanced Breast Cancer at Diagnosis: Over One Third Adherent to Screening Recommendations

**DOI:** 10.1089/whr.2020.0044

**Published:** 2020-08-31

**Authors:** Orli Friedman-Eldar, Douglas Zippel, Helit Guy-Chen, Shlomi Eitan Gur, Noa Ben-Baruch, Eran Sharon, Tanir M. Allweis

**Affiliations:** ^1^ Department of Surgery and Sarah Markowitz Breast Health Center, Kaplan Medical Center, Rehovot, Israel.; ^2^ Meirav Breast Unit, Sheba Medical Center, Ramat-Gan, Israel.; ^3^ Sackler School of Medicine, Tel Aviv University, Tel Aviv, Israel.; ^4^ Department of Plastic Surgery, Kaplan Medical Center, Rehovot, Israel.; ^5^ Department of Oncology, Kaplan Medical Center, Rehovot, Israel.; ^6^ Breast Surgery Unit, Rabin Medical Center, Petach-Tikva, Israel.; ^7^ Hebrew University Medical School, Jerusalem, Israel.

**Keywords:** advanced breast cancer, screening adherence, screening mammography

## Abstract

***Background:*** Advanced breast cancer (ABC) at diagnosis carries a worse prognosis, and can be attributed to delay in diagnosis, failure of screening tests, or aggressive biology. Better understanding of factors related with ABC at diagnosis could help decrease the proportion of such cases.

***Patients and Methods:*** This is a retrospective study of all patients diagnosed and treated for breast cancer (BC) at a single institution between 2012 and 2015. Data were collected from medical records and phone interviews, and included demographic, clinical, and tumor-related data, and adherence to screening recommendations.

***Results:*** Of 555 newly diagnosed BC patients, 390 (70.3%) were diagnosed early (stage 0–IIa), and 165 (29.7%) were diagnosed with ABC (stage IIb–IV). Of the165 patients diagnosed with ABC, 57 (34.5%) underwent screening mammography as recommended. More patients with ABC were <50 years (29.1% vs. 19%, *p* = 0.006). ABC was associated with higher grade, higher proliferation rate, Her2/neu overexpression, luminal B-like, and triple negative phenotypes. Mammography within 30 months of diagnosis was more prevalent among those diagnosed early (64.6% vs. 34.5%, *p* = 0.003). Only 31 (18.8%) of the screening eligible patients who were diagnosed at advanced stage did not adhere to screening recommendations.

***Conclusions:*** ABC at diagnosis is related to aggressive tumor biology and age <50 years. It is also associated with lower adherence to screening mammography; however, more than one third of patients diagnosed with ABC who were eligible for screening underwent screening mammography as recommended. Further research is needed to elucidate factors related with ABC at diagnosis, review screening guidelines, and develop more effective screening modalities.

## Introduction

Breast cancer (BC) may be diagnosed due to clinical symptoms or through routine screening tests; either way the stage at diagnosis may be early (stage 0–IIa) or advanced (stage IIb–IV). The latter group has lower cure rates and a worse prognosis.^1^

In Israel, according to the national cancer registry, between the years 2001 and 2010, as many as 30% of newly diagnosed BC patients presented with advanced disease, either with local or systemic spread.^2^ In contrast, compliance with screening mammography guidelines in Israel (mammography every 2 years between ages 50 and 74 years) is around 75%.^3^ It, therefore, appears that there is a subgroup of patients for whom advanced stage at diagnosis cannot be attributed to noncompliance. Possible reasons may include being outside the target screening population (*e.g.*, due to age), having a false negative result on screening, or developing an “interval cancer” between two rounds of scheduled screening, due to aggressive tumor biology.

Several studies investigated the characteristics of patients diagnosed with advanced-stage BC. One study from New Zealand (where the screening recommendations are similar to those in Israel) showed that out of 12,390 newly diagnosed BC patients between the years 2000 and 2013, 18% presented with advanced disease. Advanced stage at diagnosis in this study was significantly associated with age <40 or >70 years, lower socioeconomic status, rural residency, treatment at public hospitals, and worse tumor biology.^4^ A systematic review and meta-analysis of >860,000 patients showed that patients residing in rural areas were more likely to be diagnosed with more advanced breast cancer (ABC), compared with urban BC patients.^5^ Downing et al. found that women living in more deprived areas were more likely to be diagnosed with stage III or IV disease.^6^

Taplin et al. retrospectively studied BC patients 50 years of age and older, and found that lack of screening was more common in women with advanced-stage BC, compared with early-stage disease. Among those with advanced BC at diagnosis, women were more likely to be in the unscreened group if they were ≥75 years, unmarried, without a family history of BC, less educated, or with lower annual income.^7^

The aim of this study was to characterize women presenting with advanced BC at the time of diagnosis compared with women diagnosed with early BC in terms of the following:

1. Screening eligibility, awareness, and adherence.
2. Demographic characteristics.
3. Tumor biology.

## Patients and Methods

This retrospective observational study included women diagnosed for the first time with BC between the years 2012 and 2015 at Kaplan Medical Center, Rehovot, Israel. Patients were divided into two main groups: early BC (stage 0–IIa by AJCC classification eighth edition, 2016) and locally advanced or metastatic BC (stage IIb–IV).^8^

We included patients with ductal or lobular invasive carcinoma or ductal carcinoma *in situ*, or any of the aforementioned combination. Since our intent was to address the performance of screening mammography in women with average BC risk, we excluded women at high risk such as women with a personal history of breast or ovarian cancer, high risk lesion in the past (atypical ductal hyperplasia [ADH], atypical lobular hyperplasia [ALH], or lobular carcinoma *in situ* [LCIS]), a known breast cancer susceptibility gene mutation, or a history of mantle radiation. We also excluded males, and women with noncarcinoma breast neoplasms (*e.g.*, malignant phylloides or lymphoma of the breast).

For eligible women, data were collected from electronic files, and completed when possible by phone interviews. Data collected included demographic variables, clinic-pathological variables, medical history, family history of breast or ovarian cancer, and awareness of and adherence to screening tests.

The primary outcome was adherence to current mammography screening guidelines (every other year between the ages of 50 and 74 years) before diagnosis. Secondary outcomes included association between stage at diagnosis and tumor biology, demographic differences between both groups, and reasons for screening nonadherence in screen eligible patients.

The study was approved by the institutional review board.

### Data analysis

Statistical analysis was performed using SAS version 9.4. Continuous variables were presented using average and standard deviation, and categorical variables were presented by percentage and number (%, *n*). Tables 1–3 present the logistic regression model and the computed odds ratio. In Table 2, the chi square test was used for the following parameters: grade, size, and lymphatic spread. *p*-Values of <0.05 were considered statistically significant.

**Table 1.** Demographic Characteristics

	Advanced BC (stage IIb–4), N (%)	Early BC (stage 0–IIa), N (%)	OR (95% CI)	p* (*p < 0.05)
Mean age (years)	60.6	61.8	—	0.38
Age by decades	*N* = 165	*N* = 390		
≤30	3 (1.8)	2 (0.5)	4.2 (0.6–26.4)	0.12
31–40	16 (9.7)	18 (4.6)	2.5 (1.1–5.4)	0.02
41–50	29 (17.6)	54 (13.9)	1.5 (0.8–2.7)	0.17
51–60	33 (20.0)	93 (23.9)	1.0	Ref
61–70	37 (22.4)	125 (32.0)	0.8 (0.4–1.4)	0.51
71–80	25 (15.2)	71 (18.2)	0.9 (0.5–1.8)	0.98
>80	22 (13.3)	27 (6.9)	2.2 (1.1–4.5)	0.01
Education	*N* = 66	*N* = 216		
Years of schooling	12.8	13.0	—	0.64
Family status	*N* = 119	*N* = 313		
Married	84 (70.6)	225 (71.9)	0.7 (0.3–1.3)	0.28
Unmarried	35 (29.4)	88 (28.1)	1.0	ref
Descendants	*N* = 133	*N* = 313		
≥1	125 (93.9)	296 (94.5)	0.8 (0.3–2.1)	0.80
Occupation	*N* = 139	*N* = 335		
Employed	68 (48.9)	178 (53.1)	0.9 (0.6–1.3)	0.64
Unemployed	12 (8.6)	17 (5.1)	1.6 (0.7–3.7)	0.2
Retired	59 (42.5)	140 (41.8)	1.0	Ref
Average SES	5.82	5.91		0.33
Residence	*N* = 160	*N* = 379		
Urban	133 (83.1)	306 (80.7)	1.1 (0.7–1.9)	0.51
Medical history				
Smoking	22 (13.3)	44 (11.2)	1.2 (0.7–2.1)	0.43
Heart disease	18 (10.9)	36 (9.2)	1.2 (0.6–2.2)	0.45
Other cancer	17 (10.3)	40 (10.2)	1.0 (0.5–1.9)	0.87
Depression	21 (12.7)	46 (11.7)	1.1 (0.6–1.9)	0.68
Dementia	11 (6.6)	8 (2.0)	3.2 (1.2–8.3)	0.01
High PS	119 (72.1)	313 (80.2)	0.3 (0.2–0.6)	0.0006
Chronic diseases	*N* = 152	*N* = 371		
None	46 (30.3)	123 (33.2)	0.8 (0.5–1.4)	0.64
One	42 (27.6)	100 (26.9)	1.0 (0.6–1.6)	0.90
Two or more	64 (42.1)	148 (39.9)	1.0	Ref
Family history of BC	*N* = 138	*N* = 367		
None	88 (63.8)	225 (61.3)	1.1 (0.6–2.0)	0.66
First-degree relative	32 (23.2)	89 (24.3)	1.0 (0.5–2.0)	0.86
Not first-degree relative	18 (13.0)	53 (14.4)	1.0	Ref
Prior breast surgery or biopsy	*N* = 97	*N* = 300		
	23 (23.7)	66 (22.0)	1.1 (0.6–18)	0.72
	*N* = 44	*N* = 135		
BRCA mutation^a^	5 (11.3)	5 (3.7)	3.3 (0.9–12)	0.06

^a^ Tested after BC diagnosis.

BC, breast cancer; BRCA, breast cancer genes 1/2; OR, odds ratio; PS, performance status; SES, socioeconomic status, address based (1–10).

**Table 2.** Tumor Characteristics

	Advanced BC (stage IIb–4), N = 165 (%)	Early BC (stage 0–IIa), N = 390 (%)	OR (95% CI)	p* (*p < 0.05)
Stage at diagnosis			NR	NR
0	0 (0.0)	97 (24.9)		
1	0 (0.0)	180 (46.2)		
2A	0 (0.0)	113 (28.9)		
2B	79 (47.9)	0 (0.0)		
3	62 (37.6)	0 (0.0)		
4	24 (14.5)	0 (0.0)		
Tumor size (T)				NR
T0	2 (1.2)	37 (9.5)		
T1	6 (3.6)	225 (57.7)		
T2	70 (42.5)	123 (31.5)		
T3	49 (29.7)	2 (0.5)		
T4	37 (22.4)	0 (0.0)		
Tx	1 (0.6)	3 (0.8)		
Lymphatic spread (N)				NR
N0	39 (23.6)	370 (94.9)		
N1	111 (67.3)	18 (4.6)		
N2	10 (6.1)	0 (0.0)		
N3	4 (2.4)	0 (0.0)		
Nx	1 (0.6)	2 (0.5)		
Histology				
DCIS	0 (0.0)	97 (24.8)	—	0.94
IDC	146 (88.5)	262 (67.2)	0.8 (0.1–5.0)	0.85
ILC	17 (10.3)	28 (7.2)	0.9 (0.1–6.2)	0.95
IDC+ILC	2 (1.2)	3 (0.8)	1.0	Ref.
Grade	*N* = 153	*N* = 380		
1	19 (12.4)	160 (42.1)	0.3 (0.1–0.5)	<0.0001
2	55 (35.9)	154 (40.5)	1.0	Ref.
3	79 (51.6)	66 (17.4)	3.3 (2.1–5.2)	<0.0001
IHC pattern				
ER+	118 (71.9)	353 (91.4)	0.2 (0.1–0.3)	<0.0001
PR+	88 (53.6)	305 (79.2)	0.3 (0.2–0.4)	<0.0001
HER2+	36 (21.9)	37 (12.5)	1.9 (1.1–3.2)	<0.0001
67Ki	*N* = 152	*N* = 293		
Weak (<25)	67 (44.1)	214 (73)	1.0	Ref.
Intermediate (25–50)	72 (47.4)	71 (24.3)	4.0 (2.3–6.8)	0.0001
Strong (>50)	13 (8.5)	8 (2.7)	6.5 (2.4–17.2)	0.0002
Subtype	*N* = 164	*N* = 290		
Luminal A like	65 (39.6)	212 (72.3)	1.0	Ref.
Luminal B like	34 (20.7)	25 (8.5)	4.4 (2.4–7.9)	<0.0001
TNBC	30 (18.3)	18 (6.1)	5.4 (2.8–10.3)	<0.0001
TP/HER2+	35 (21.4)	38 (12.9)	3.0 (1.7–5.1)	<0.0001

BC, breast cancer; DCIS, ductal carcinoma *in situ*; ER, estrogen receptor; HER-2, human epidermal growth factor receptor 2; IDC, invasive ductal carcinoma; IHC, immunohistochemistry; ILC, invasive lobular carcinoma; N, node; NR, not relevant; PR, progesterone receptor; Ref., reference; T, tumor; TNBC, triple-negative breast cancer; TP, triple-positive.

**Table 3.** Compliance with Screening Recommendations

	Advanced BC (stage IIb–4), N (%)	Early BC (stage 0–IIa), N (%)	OR (95% CI)	p* (*p < 0.05)
Awareness of screening recommendations^a^	*N* = 62	*N* = 196		
	54 (87.0)	175 (89.2)	0.8 (0.3–1.9)	0.63
	*N* = 165	*N* = 390		
				
Screening adherence^b^				
Targeted population	41 (24.8)	197 (50.5)	0.1 (0.05–0.3)	0.0001
Untargeted population	16 (9.7)	55 (14.1)	0.2 (0.08–0.8)	0.02
Screening inadherence				
Targeted population	31 (18.8)	59 (15.2)	0.4 (0.1–1.2)	0.14
Untargeted population	66 (40.0)	71 (18.2)	2.2 (1.3–3.9)	0.003
Missing data	11 (6.7)	8 (2)	—	—
No previous mammography	*N* = 147	*N* = 367		
Diagnosed <50 years old	31 (21.0)	34 (9.2)	4.8 (2.6–8.5)	<0.0001
Diagnosed >50 years old	30 (20.4)	39 (10.6)	3.5 (2.0–6.1)	<0.0001
Reason for inadherence^c^	*N* = 61	*N* = 91		
Missing data	36 (59.0)	58 (63.7)	0.6 (0.1–2.0)	0.43
Fear/neglect	11 (18.0)	5 (5.5)	2.2 (0.4–10.3)	0.31
Lack of awareness	5 (8.2)	10 (11.0)	0.5 (0.1–2.3)	0.38
Did not receive invitation	3 (4.9)	7 (7.7)	0.4 (0.07–2.5)	0.34
Poor general condition	6 (9.9)	6 (6.6)	1.0	Ref.
Other	0 (0.0)	5 (5.5	—	—
Routine CBE before Dx	15 (9.0)	102 (26.1)	0.3 (0.1–0.6)	0.001
Routine BSE before Dx	26 (15.7)	112 (28.7)	0.5 (0.2–0.9)	0.02

^a^ Knowledge of which test the screening program includes, the age to start, and the frequency recommended.

^b^ Adherence considered as screening test within 30 month before Dx. Targeted population is of ages 50–74 years.

^c^ Calculated only for women >50 years who did not undergo screening according to recommendations.

BSE, breast self-examination; CBE, clinical breast examination; Dx, diagnosis.

## Results

A total of 555 newly diagnosed BC patients fulfilled the inclusion criteria. Of these, 390 women (70.3%) were diagnosed with early BC, and 165 (29.7%) had advanced disease at the time of diagnosis.

**FIG. 1.**
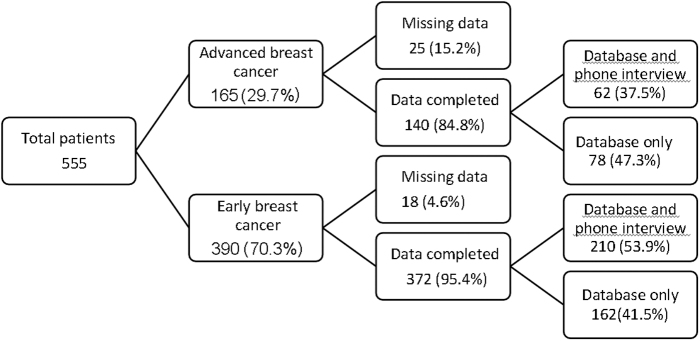
Distribution of data completeness in the different groups.

Owing to the retrospective nature of this study, some data could not be determined (Fig. 1). Missing data were defined as lack of information regarding date of the last mammogram before BC diagnosis. Data extracted from electronic patient files such as those related to tumor biology were complete, but clinical and demographic variables as well as information regarding screening awareness and reason for screening inadherence were often lacking in the medical records and had to be collected through phone interviews, which were not always informative, due to recollection difficulties, patient unavailability, or demise. Of the 25 (15.2%) women with missing data in the advanced-stage group, 13 had died, 3 refused the phone interview, and 9 could not be reached, whereas in the early-stage group data were missing for 18 (4.6%) patients, 2 of whom had died, 4 refused the phone interview, and 8 could not be reached.^*^

Table 1 presents the demographic characteristics of the study population. The average age was similar in both groups, but among women diagnosed with advanced BC, there were significantly more women <40 or >80 years, compared with the early-stage group (9.7% vs. 4.6%, *p* = 0.02, and 13.3% vs. 6.9%, *p* = 0.01, respectively). We found that dementia was significantly more frequent among patients diagnosed with advanced-stage disease (6.6% vs. 2.0%, *p* = 0.01). High-performance status (defined as no mobility limitations or dependency on others for daily life activities), was more prevalent among patients diagnosed with early-stage disease (80.2% vs. 72.1%, *p* = 0.0006). We did not find significant differences for other chronic conditions such as depression, although it is a parameter that may be under-reported. Jewish ethnicity and Ashkenazi origin were distributed equally between the ABC group and the early diagnosis group (95.2% vs. 95.8%, p = 0.6, and 60.1% vs. 53.7%, p = 0.7, respectively). We did not detect any association between stage at diagnosis and education, family, or socioeconomic status, perhaps due to relative small numbers or missing data.

Prior history of breast surgery or breast biopsy was not associated with stage at diagnosis, contrary to the expectation that women with such a history would have heightened awareness and closer follow-up. Nor was a family history of BC associated with stage at diagnosis.

Genetic testing was done for around one third of the patients after BC diagnosis. Previously unknown breast cancer genes 1/2 (BRCA) mutations were more common among women diagnosed with advanced-stage BC, although due to the small numbers this was not statistically significant (11.3% vs. 3.7%, *p* = 0.06). Overall, 10 of those tested were found to be BRCA mutation carriers, 8 of them BRCA1.

Table 2 summarizes the biological and histological characteristics of the tumors, and clinical staging at the time of diagnosis. Estrogen receptor positive tumors were classified as “luminal A like” if the progesterone receptor (PR) was also positive and histological grade was I or II. If PR was negative and/or the histological grade was high, they were classified as “luminal B like.”

Histological type was distributed similarly among both groups, except for pure ductal carcinoma *in situ*, which by definition is early-stage disease (Table 2). Tumors of patients diagnosed with advanced BC were at higher grade, higher proliferation rate as determined by Ki67 immunohistochemistry, more often Her2/neu overexpressing, and with less favorable receptor profile, that is, nonluminal-A like subtype (60.2% vs. 27.5%, *p* < 0.0001) or triple negative cancers.

Women were considered adherent to screening guidelines if they started undergoing routine mammography at 50 years, and had had one within 30 months preceding BC diagnosis, with 30 months chosen so as to allow for small deviations not to be counted as screening inadherence.

Adherence to screening in the targeted population reduced the risk of advanced BC to a rate of 41/238 (17.2%), whereas for the targeted nonadherent group the rate of ABC was 31/90 (34.4%) (Table 3).

**FIG. 2.**
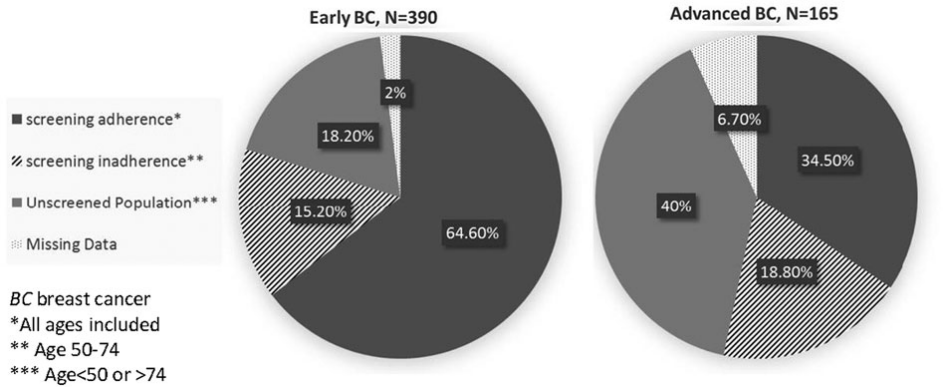
Adherence with screening mammography recommendations. BC, breast cancer.

Among women diagnosed with advanced BC there were twice as many women >50 years who had never had a previous mammogram compared with those diagnosed early (20.4% vs. 10.6%, *p* < 0.0001), as seen in Table 3. One third of women with advanced BC had screening mammography within 30 months before their diagnosis (Fig. 2). Of the 57 women in this group, only 13 (22.8%) were diagnosed after a routine screening test, and the other 44 (77.2%) were diagnosed after a clinical symptom, which appeared between two rounds of screening tests (“interval cancer”).

## Discussion

Several randomized trials showed that screening for BC decreases mortality from the disease and improves the chances for long-term cure.^9^ In a publication of the Israeli Ministry of Health in 2015, >20% of newly diagnosed BC patients were <50 years.^10^ This is a significant portion of women for whom there is no recommended screening test for early BC diagnosis.

In this study, nearly one third of the newly diagnosed BC patients had advanced disease at diagnosis. Adherence to screening in the targeted population reduced the risk of advanced BC by half, compared with the targeted nonadherent group. However, only 18.8% of patients diagnosed with ABC were noncompliant with screening recommendations. Another 49.7% of the women in the ABC group were nontargeted population (<50 or >75 years of age), either screened or unscreened, as reflected in the age distribution differences between the groups. Screening mammography clearly reduces the likelihood of diagnosis at an advanced stage, but still there is a substantial portion of women who present with advanced disease even though they followed the screening guidelines, so revision of the current guidelines should be considered. If the screening mammography guidelines included patients from the age of 40, it would have made 29 more cases in our series potentially detectable. In contrast, 16 untargeted women were screened and still developed ABC. On top of that we must take into account that additional screening in groups that are of low overall risk carries potential harms, such as false positive results, overdiagnosis, and radiation exposure.

About one third (34.5%) of women diagnosed with ABC underwent a screening test (including patients who were not within the targeted screening guidelines based on age). This is the group for whom screening failed to bring about early diagnosis. (“Interval cancer”), either due to a false negative screening test or aggressive tumor biology. The later was shown in this study to be significantly more prevalent in the ABC group compared with the early diagnosed group.

It is hard to ascertain the reasons behind noncompliance with screening recommendations in this type of study. No significant difference was detected between both groups in awareness of screening recommendations.

Women who were diagnosed with early BC were more likely to undergo routine clinical breast examination and occasional breast self-examination, practices that have been shown to be ineffective in decreasing BC associated mortality or enhancing early diagnosis.^11–16^ It may be that these women are more compliant with medical recommendations in general and, therefore, more likely to undergo screening tests more vigilantly.

We found dementia and low performance status were significantly associated with advanced-stage disease, but no significant differences for other chronic conditions such as depression, although depression may be under-reported.

Many studies found an association between lower socioeconomic status and lower education level with advanced BC at diagnosis.^3–5^ In this study, we did not detect any association between stage at diagnosis and economical or family status, or urban versus rural residence, possibly due to small numbers or missing data.

Prior history of breast surgery or biopsy was not associated with stage at diagnosis, nor was a family history of BC, contrary to the expectation that these women would have heightened awareness and closer surveillance. It is possible that for some women such a history is an impediment to screening due to fear, repression or denial, as seen in previous studies.^17^

Ten BRCA mutation carriers were detected among 179 patients tested after being diagnosed with BC, 8 of them were BRCA1, in keeping with the established tendency of BRCA1 related tumors to be more aggressive.^18,19^ The two BRCA2 carriers were in the early-diagnosed group.

We acknowledge that this study has several limitations. Owing to the retrospective nature of this study, some data could not be determined. Therefore, no conclusions can be drawn regarding reason for screening inadherence. Data were missing more often for the ABC group (15.2 vs. 4.6%), possibly related to a higher mortality rate in this group.

## Conclusions

To provide BC patients with the best possible prognosis, early diagnosis is paramount. Improving awareness of and compliance with screening mammography may increase early diagnosis of BC, but it is not clear whether screening mammography alone can sufficiently decrease the rate of patients with ABC at the time of diagnosis. In our study, as many as 75% of patients with ABC at diagnosis were either adherent with screening recommendations or nontargeted population, which is a substantial limitation of screening mammography. Redefinition of screening mammography guidelines should be considered, such as extending the guidelines to include women <50 years. Also, efforts should be made to establish new screening tests, imaging based and others, to complement or replace routine screening mammography as is currently practiced. Diagnosis of BC at an advanced stage was also found to be associated with dementia and low performance status.

## Abbreviations Used

ABC: advanced breast cancer
BC: breast cancer
BRCA: breast cancer genes 1/2
ER: estrogen receptor
OR: odds ratio
PR: progesterone receptor

## Notes

^*^ From here on all percentages relate to the subgroups (early or advanced).
